# Validating Age-Equitable Mixed Emotion Film Stimuli for Lifespan Research

**DOI:** 10.1093/geroni/igaf122.4302

**Published:** 2025-12-31

**Authors:** Ryan Muskin, Lucas Hamilton, Michelle Moore, Eric Allard

**Affiliations:** Cleveland State University, Cleveland, Ohio, United States; Augustana University, Sioux Falls, South Dakota, United States; Cleveland State University, Cleveland, Ohio, United States; Cleveland State University, Cleveland, Ohio, United States

## Abstract

Mixed emotions are increasingly recognized as important for well-being and adaptive functioning in later life. However, research on age differences in mixed emotions is limited by methodological challenges, particularly the lack of age-equitable emotion elicitors. Theoretical frameworks suggest that older adults may experience less intense affective blends than younger adults, raising concerns that commonly used stimuli (e.g., film clips) may not evoke comparable responses across the lifespan . This study tested whether specific film genres can elicit more age-equitable emotional responses, thereby reframing how mixed emotions are studied across adulthood. Participants Participants were younger and older adults recruited via Prolific who viewed one of three clips: a dark comedy (Barry, N = 258), a biographical drama (King Richard, N = 185), or a fantasy-action film (Avengers: Endgame, N = 185). Age (Younger, Older) × Time (Pre, Post) ANOVAs revealed significant age × time interactions for Barry and Avengers (p < .01; ηp² = .03–.05), with older adults reporting more diminished positive affect. However, King Richard produced no significant age differences, and item-level analyses showed age equivalence in high-intensity positive and negative affect across King Richard and Avengers. Across clips, younger adults reported higher mixed emotions overall, but findings suggest genre and intensity modulate age-related differences. These results support theoretical predictions about mixed emotion intensity while offering a novel approach to designing emotionally evocative stimuli that work equitably across age groups. By identifying affective blends that transcend age-related response biases, this study advances methodological practices that more accurately represent emotional experience across the lifespan.

